# A Cost Variation Analysis of Drugs Available in the Indian Market for the Management of Thromboembolic Disorders

**DOI:** 10.7759/cureus.7964

**Published:** 2020-05-05

**Authors:** Avik Ray, Ahmad Najmi, Gaurav Khandelwal, Balakrishnan Sadasivam

**Affiliations:** 1 Pharmacology, All India Institute of Medical Sciences Bhopal, Bhopal, IND; 2 Cardiology, All India Institute of Medical Sciences Bhopal, Bhopal, IND

**Keywords:** anticoagulants, antiplatelets, cost variation, fibrinolytics, indian market, pharmacoeconomics, drug pricing policy

## Abstract

Introduction

Cardiovascular diseases (CVDs) have become one of the major causes of mortality among the Indian population. The costs of anticoagulant, antiplatelet, and fibrinolytic drugs that are used to treat various thromboembolic disorders and used as prophylactics for individuals at high risk of CVDs vary widely in the Indian pharmaceutical market. The aim of this study was to evaluate the cost variation of different brands of drug formulations and to compare the branded prices of the formulations with their corresponding generic and ceiling prices.

Materials and methods

This study followed an analytical method. Costs of various drugs were obtained from the October - December 2019 edition of the Current Index of Medical Specialities (CIMS) and December 2019 edition of the Monthly Index of Medical Specialities (MIMS) India. Cost ratio and percentage variation in cost per tablet/capsule/injection of different drugs available in the Indian market and manufactured by different pharmaceutical companies were calculated. Comparison of the branded prices with generic and ceiling prices was also performed for different drugs by using information available from official websites.

Results

Percentage variation in cost among the commonly prescribed drugs for the management of thromboembolic disorders was found to be highest for prasugrel 10 mg tablet (1,408.44%) while it was lowest for fondaparinux 2.5 mg / 0.5 ml injection (20%). Among the commonly prescribed drugs that are under Drugs Prices Control Order (DPCO) price control, streptokinase 1.5 MIU injection had the highest cost variation (132.02%) while enoxaparin 60 mg / 0.6 ml injection had the lowest (4.99%). Among some of the important formulations under the Jan Aushadhi scheme (JAS), acenocoumarol 2 mg tablet had the highest cost variation (680.09%) and cilostazol 50 mg tablet had the lowest (55.46%).

Conclusions

Wide differences exist in the costs of various anticoagulants, antiplatelets, and fibrinolytics available in the Indian market. The prescribing physician should be aware of theses variations and prescribe medicines accordingly, keeping in mind the financial status of the patients.

## Introduction

Cardiovascular diseases (CVDs) have become the major cause of mortality in India [1]. As compared to the residents of the European countries, CVD affects the Indians at least a decade earlier, in the most productive years of their lives [2]. In India, 52% of the deaths due to CVD occur before the age of 70 years as compared to 23% in the Western populations [3]. According to an estimation of the World Health Organization (WHO), India lost approximately $237 billion owing to loss of productivity and healthcare expenses due to CVDs over a period of 10 years (2005-2015) [4]. Drugs for thromboembolic disorders, such as antiplatelets, can reduce the incidence rate of cardiovascular events by 20-25% in patients with either established CVD or those who are at high risk of one [5]. Anticoagulants, antiplatelets, and fibrinolytics are the main drugs used to treat thromboembolic disorders. They are used for secondary prophylaxis in a patient who has had a thromboembolic event or as primary prophylaxis for patients at high risk of CVDs.

In developing nations like India, drug prices play a pivotal role in healthcare. Besides safety and efficacy, drug prices are considered while including drugs in formularies and the National List of Essential Medicines (NLEM). Rational prescribing consists of prescribing the right drug at the right dose and right formulation for the right duration and at the right price, which the concerned patient can afford. With the rapid growth of the Indian pharmaceutical landscape, the national market is full of branded generics manufactured by various firms with a lot of variations in the prices of different brands of the same formulation. There has been a worrying lack of consideration of the difference in the prices of various brands of drugs among the physicians. This has resulted in an increase in the overall healthcare expenditures and has affected the financial status of the patients immensely, especially those with CVDs who require chronic treatment. In India, the out-of-pocket (OOP) expenditure as a percentage of the total health expenditure has remained consistently high: 69.4% in 2004, 64.2% in 2014, and 62.6% in 2015 [6-8].

The National Pharmaceutical Pricing Authority (NPPA) was established on August 29, 1997, to regulate the prices of pharmaceutical drugs in India. The implementation of the National Pharmaceutical Pricing Policy, 2012, and the Drugs Prices Control Order (DPCO), 2013 was brought about by NPPA [9]. It safeguards the interest of both the manufacturer and the consumers by ensuring the availability of essential medicines at affordable prices. It fixed the ceiling prices of 330 formulations of medicines mentioned in the NLEM, 2015 [9]. With the cost of the raw materials increasing steeply, the NPPA has recently hiked the prices of 21 drug formulations by 50% [10]. The Government of India has allowed a price hike of 15-20% for drugs re-launched with innovations like reduced adverse events or more compliant administration techniques such as skin patches to incentivize the firms to pursue innovation with more focus [11].

To increase the availability of generic medicines to the population, the Government of India launched the Jan Aushadhi Scheme (JAS) in 2008 [12]. Generic drug stores were opened across the nation to provide affordable medicines to the masses. However, most of those stores have become non-functional due to various issues such as lack of support from the state government, flawed supply chain and its poor management, non-prescription of generic medicines, poor perspectives, and lack of awareness. A study has shown that with the drug prices under the JAS scheme, the OOP expenditure on medicines in India is highly unlikely to decline [12]. The study, however, did not include drugs used for thromboembolic disorders.

Currently, very few studies on the cost variation analysis of anticoagulants, antiplatelets, and fibrinolytics available in the Indian market along with a direct comparison with the ceiling price and the generic price under the JAS scheme are available in the literature. Our study had the following objectives: A) to evaluate the variation in the prices of different brands of the same drug by obtaining the percentage cost variation and B) to compare the branded price of the formulations of different drugs with their corresponding ceiling price and generic price.

## Materials and methods

Study type

This was an analytical-type study. The cost of different branded and generic formulation of drugs, both oral and systemic, were calculated for each unit or tablets or capsules or injections. Various formulations of anticoagulants, antiplatelets, and fibrinolytics were included in the study to compare the prices for the same strength and route of administration. Drugs manufactured by a single company and fixed-dose combinations (FDCs) were also included.

Study materials

The October - December 2019 edition of the Current Index of Medical Specialities (CIMS) and the December 2019 edition of Monthly Index of Medical Specialities (MIMS) India were referred for maximum and minimum prices of the drugs under consideration in Indian rupees (INR) as available in the Indian market. Drugs and formulations whose prices were not mentioned in the above sources were excluded from the study. To compare the prices of branded drugs with the corresponding generics, the prices of the generic formulations as given on the official websites of the Bureau of Pharma PSUs of India (BPPI) and the Government of India were used [13].

Definitions

The Defined Daily Dose (DDD) is the assumed average maintenance dose of a drug used for a particular indication in adults. Cost in terms of minimum, maximum, and median cost per DDD have been calculated besides per unit calculation. Cost ratio is the ratio of the cost of the costliest to the cheapest branded formulations of the same drug, which tells us by how many times the cost of the most expensive drug is higher than the cheapest one for each of the drugs considered for evaluation. Percentage cost variation was calculated as follows: (Maximum branded price of a particular drug formulation: Minimum branded price of the same drug formulation) / Minimum branded price of the same drug formulation X 100. Price per DDD was calculated as follows: price per unit X DDD.

Statistical analysis

The data obtained from the mentioned sources were analyzed using Microsoft Excel® 2019 software (Microsoft, Redmond, WA). The price variations have been expressed in percentages and the results have been shown in tables, bar charts, and a scatter plot.

## Results

Cost variation of the drugs

**Figure 1:**
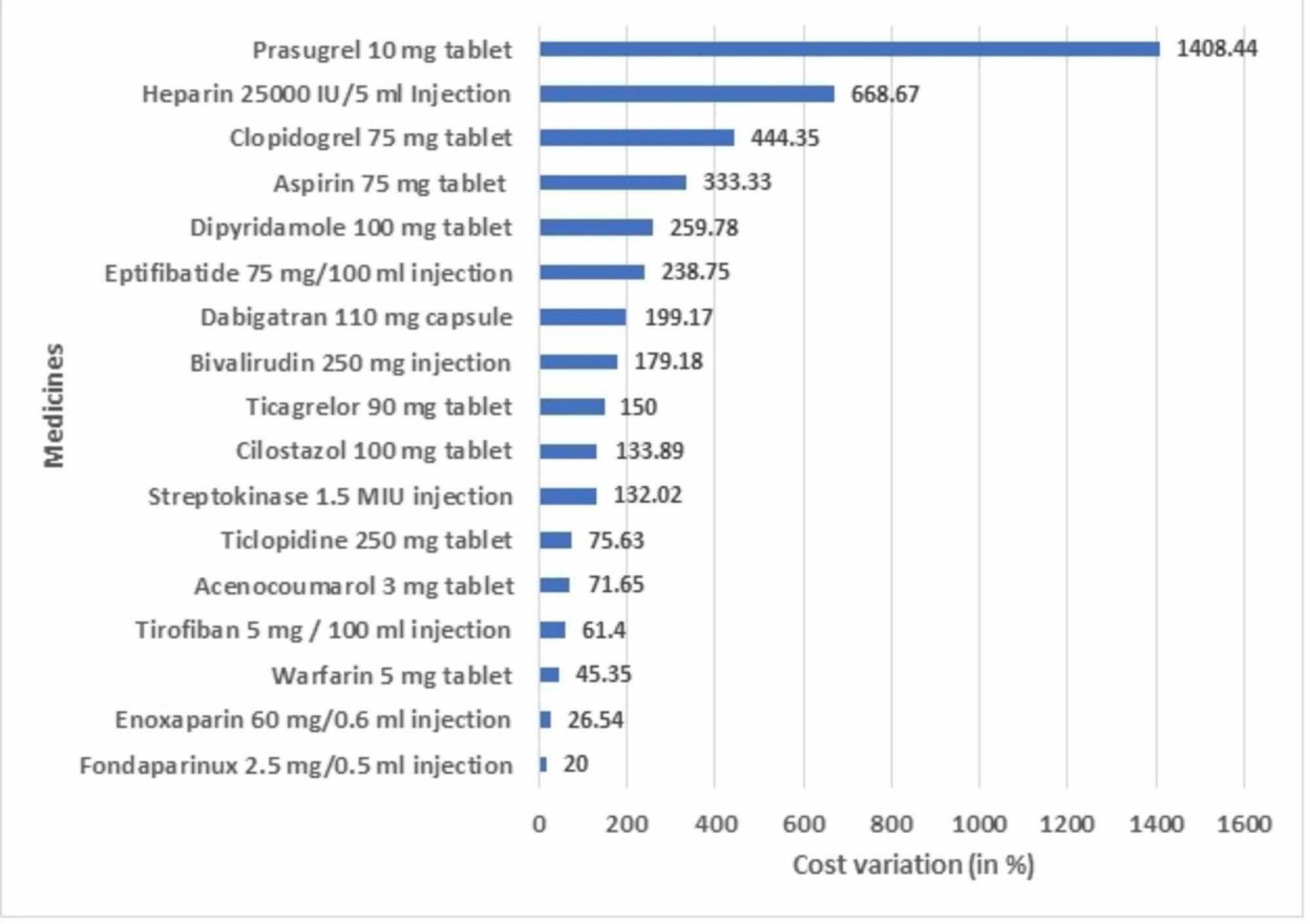
Comparison of the cost variation of commonly prescribed drugs for the management of thromboembolic disorders MIU: million international unit

The prices of various antiplatelets, anticoagulants, and fibrinolytics available in the Indian market and produced by different pharmaceutical companies were analyzed. Tables 1-3 depict their cost ratio and cost variation. Wide variations in the prices of different brands of the same drugs existed, both in the NLEM and Non-National List of Essential Medicines (NNLEM) categories. We have mainly analyzed single drugs except for two combinations: aspirin + clopidogrel and aspirin + prasugrel. Among all the drugs used for treating thromboembolic disorders, the highest percentage of cost variation was observed for prasugrel 5 mg tablets (2,455.55%). Among other single drugs, cost variations were notably large as well; for example, prasugrel 10 mg tablet (1,408.44%), heparin 25,000 IU / 5 ml injection (668.67%), aspirin 325 mg tablet (585.18%), clopidogrel 75 mg tablet (444.35%), aspirin 100 mg tablet (435.13%), dipyridamole 25 mg tablet (390.77%), and dabigatran 75 mg capsule. Among the drugs having more than one brand available in the Indian market, the lowest cost variation was observed for aspirin 75 mg + prasugrel 10 mg capsule (4.1%) followed by aspirin 50 mg tablet (5%), fondaparinux 7.5 mg / 0.6 ml injection (9.85%), enoxaparin 60 mg / 0.6 ml and 40 mg / 0.4 ml injection (26.54% and 28.72% respectively) (Figure 1).

**Table 1:** Cost variation among anticoagulants *Denotes selective imposition of price ceiling. ^†^Denotes under DPCO price ceiling. ^‡^Denotes not under DPCO price ceiling DDD: daily defined dose; TU: tuberculin unit; IU: international unit, INR: Indian Rupees; DPCO: Drugs Prices Control Order

S.no.	Drug	DDD	Dose and formulation (no. of brands)	Minimum price per unit, INR	Minimum price per DDD, INR	Maximum price per unit, INR	Maximum price per DDD, INR	Cost ratio	Cost variation, %
1.	Acenocoumarol	5 mg	1 mg tablet (6)	2.75	13.75	5	25	1.82	81.82
			2 mg tablet (6)	3.9	9.75	11.43	28.57	2.93	193.08
			3 mg tablet (5)	8.5	14.19	14.59	24.36	1.72	71.65
			4 mg tablet (6)	5.7	7.12	18.5	23.12	3.24	224.56
2.	Enoxaparin^*^	2 TU (200 mg)	40 mg / 0.4 ml injection^†^ (7)	385	1,925	495.59	2,477.95	1.29	28.72
			60 mg / 0.6 ml injection^†^ (6)	475	1,567.5	601.08	1,983.56	1.26	26.54
			20 mg / 0.2 ml injection^‡^ (1)	325	3,250	325	3,250	1	0
3.	Fondaparinux	2.5 mg	2.5 mg / 0.5 ml injection (5)	655	655	786	786	1.2	20
			7.5 mg / 0.6 ml injection (3)	1,320	440	1,450	483.33	1.1	9.85
4.	Dabigatran	300 mg	75 mg capsule (4)	19.9	79.6	71.8	287.2	3.61	260.8
			110 mg capsule (6)	24	65.45	71.8	195.82	2.99	199.17
			150 mg capsule (4)	29	58	71.8	143.6	2.47	147.59
5.	Heparin	10 TU	25,000 IU / 5 ml injection (19)	45	180	345.9	1,383.6	7.69	668.67
6.	Warfarin^*^	7.5 mg	1 mg tablet^‡^ (3)	1	7.5	2.64	19.80	2.64	164.1
			2 mg tablet^‡^ (2)	2.29	8.59	3.42	12.81	1.49	49.17
			3 mg tablet^‡^ (3)	1.98	4.96	2.92	7.3	1.47	47.25
			5 mg tablet^†^ (5)	2.41	3.61	3.5	5.25	1.45	45.35
7.	Bivalirudin	250 mg	250 mg injection (6)	5,860	5,860	16,360	16,360	2.79	179.18
8.	Rivaroxaban	20 mg	20 mg tablet (1)	145	145	145	145	1	0

**Table 2:** Cost variation among antiplatelets *Denotes selective imposition of price ceiling. ^†^Denotes under DPCO price ceiling. ^‡^Denotes not under DPCO price ceiling DDD: daily defined dose; FC: film-coated; INR: Indian Rupees; DPCO: Drugs Prices Control Order

S.no.	Drug	DDD	Dose and formulation (no. of brands)	Minimum price per unit, INR	Minimum price per DDD, INR	Maximum price per unit, INR	Maximum price per DDD, INR	Cost ratio	Cost variation, %
1.	Aspirin^*^	1,000 mg	50 mg tablet^‡^ (2)	0.2	4	0.21	4.2	1.05	5
			75 mg tablet^†^ (11)	0.15	2	0.65	8.67	4.33	33.33
			100 mg tablet^†^ (3)	0.18	1.85	0.99	9.9	5.35	435.13
			150 mg tablet^†^ (9)	0.23	1.53	0.85	5.67	3.69	269.56
			325 mg tablet^†^ (3)	0.22	0.66	1.48	4.55	6.85	585.18
2.	Cilostazol	200 mg	50 mg tablet (10)	5.34	21.34	12.51	50.04	2.34	134.44
			100 mg tablet (9)	10.24	20.48	23.95	47.9	2.34	133.89
3.	Clopidogrel^*^	75 mg	75 mg tablet^†^ (62)	2.48	2.48	13.5	13.5	5.44	444.35
			75 mg FC tablet^‡^ (14)	3.8	3.8	10.22	10.22	2.69	168.95
			150 mg tablet^‡^ (4)	8.8	4.4	15.21	7.6	1.73	72.84
			150 mg FC tablet^‡^ (3)	7.38	3.69	16.88	8.44	2.29	128.73
			300 mg tablet^‡ ^(1)	9.11	2.28	9.11	2.28	1	0
			300 mg FC tablet^‡^ (2)	13	3.25	21.73	5.43	1.67	67.15
4.	Dipyridamole	400 mg	25 mg tablet (3)	0.27	4.34	1.33	21.28	4.91	390.77
			75 mg tablet (2)	0.79	4.21	3.81	20.32	4.82	382.28
			100 mg tablet (5)	1.08	4.34	3.9	15.6	3.60	259.78
5.	Eptifibatide	200 mg	20 mg / 10 ml injection (5)	1,275	12,750	2,900	29,000	2.27	127.45
			75 mg / 100 ml injection (12)	4,000	10,680	13,550	36,178.5	3.39	238.75
6.	Ticlopidine	500 mg	250 mg tablet (11)	7.55	15.1	13.26	26.52	1.76	75.63
7.	Tirofiban	10 mg	5 mg / 100 ml injection (12)	3,700	7,400	5,972	11,944	1.61	61.4
8.	Ticagrelor	180 mg	60 mg tablet (1)	40	120	40	120	1	0
			90 mg tablet (7)	12	24	30	60	2.5	150
9.	Prasugrel	10 mg	5 mg tablet (11)	5.4	10.8	138	276	25.56	2,455.55
			5 mg FC tablet (6)	9	18	12.53	25.06	1.39	39.22
			10 mg tablet (12)	9.48	9.48	143	143	15.08	1,408.44
			10 mg FC tablet (7)	15.22	15.22	24.7	24.7	1.62	62.29
10.	Aspirin + clopidogrel		50 mg + 75 mg tablet (1)	3.3	-	3.3	-	1	0
			75 mg + 75 mg tablet (34)	2.37	-	7.8	-	3.29	229.11
			75 mg + 75 mg FC tablet (11)	2	-	4.71	-	2.35	135.5
			75 mg + 75 mg capsule (12)	0.31	-	6.25	-	20.16	1,916.13
			150 mg + 75 mg tablet (23)	2.5	-	15.78	-	6.31	531.2
			150 mg + 75 mg FC tablet (8)	2.2	-	4.46	-	2.03	102.73
			150 mg + 75 mg capsule (9)	2.05	-	5.08	-	2.48	147.8
			150 mg + 150 mg tablet (1)	2.83	-	2.83	-	1	0
11.	Aspirin + prasugrel		75 mg + 10 mg capsule (2)	22.7	-	23.63	-	1.04	4.1

**Table 3:** Cost variation among fibrinolytics *Denotes selective imposition of price ceiling. ^†^Denotes under DPCO price ceiling. ^‡^Denotes not under DPCO price ceiling DDD: daily defined dose; MIU: million international unit, INR: Indian Rupees; DPCO: Drugs Prices Control Order

S.no.	Drug	DDD	Dose and formulation (no. of brands)	Minimum price per unit, INR	Minimum price per DDD, INR	Maximum price per unit, INR	Maximum price per DDD, INR	Cost ratio	Cost variation, %
1.	Streptokinase^*^	1.5 MIU	0.75 MIU injection^‡^ (15)	1181.65	2,363.3	2,178.32	4,356.64	1.84	84.34
			1.5 MIU injection^†^ (23)	1,680.08	1,680.08	3,898.09	3,898.09	2.32	132.02
2.	Urokinase	3 MIU	0.25 MIU injection (8)	1,190	14,280	2,290	27,480	1.92	92.44
			0.5 MIU injection (11)	1,752	10,512	3,799	22,794	2.17	116.84

Comparison of the costs with DPCO price ceiling

**Figure 2:**
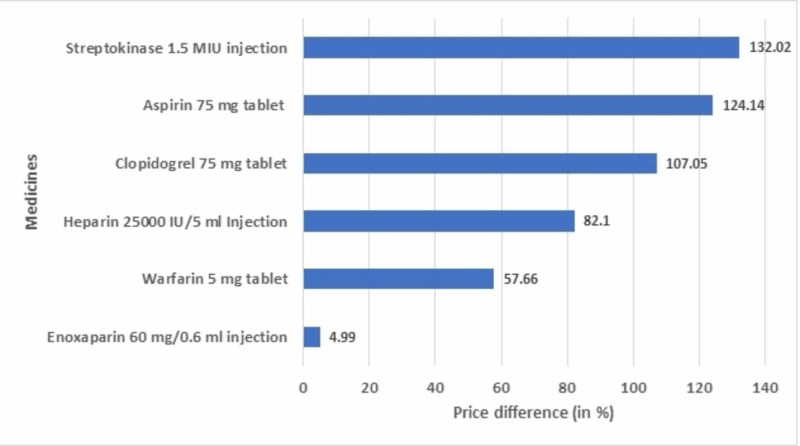
Comparison of the price variation of commonly prescribed drugs under DPCO control DPCO: Drugs Prices Control Order; IU: international unit; MIU: million international unit

On comparing the prices of the drugs under DPCO control, it was found that the maximum market price (the costliest brand of a particular drug) of all the drugs considered were higher than the ceiling prices recommended in NPPA 2018, with the highest being for aspirin 100 mg tablet (491.75%) followed by aspirin 325 mg tablet (184.62%) and with the lowest being for enoxaparin 60 mg / 0.6 ml injection (4.99%) (Figure 2).

Comparison of prices: branded versus generic

Large differences could be observed between branded and generic products of the same drug. Prices of the branded products were compared with the generic prices as given on the official website of the Bureau of Pharma PSUs of India (BPPI) (Under Department of Pharmaceuticals, Govt of India). The median branded drug price for each drug was compared with the corresponding generic price. Except for aspirin 325 mg tablet for which the median branded drug price was observed to be lower than the prescribed generic price by 33.68%, all the other drugs whose generic prices were available had a median branded price much higher than the generic price, with the highest and the lowest being for acenocoumarol 2 mg tablet (680.09%) and cilostazol 50 mg tablet (55.46%) respectively (Table 4).

**Table 4:** Comparison of prices between branded drugs and generic drugs (under the Jan Aushadhi scheme) IU: international unit, MIU: million international unit; INR: Indian Rupees

S.no.	Drug	Dose and formulation (quantity)	Minimum price (branded), INR	Median price (branded), INR	Maximum price (branded), INR	Price (generic), INR	Price difference, %
1.	Acenocoumarol	2 mg tablet (1)	3.9	8.42	11.43	1.08	680.09
2.	Enoxaparin	40 mg / 0.4 ml injection (0.4 ml)	385	409.53	495.59	163	151.24
		60 mg / 0.6 ml injection (0.6 ml)	475	552.75	601.08	180	207.08
3.	Heparin	25,000 IU / 5 ml injection (5 ml)	45	215.4	345.9	40.28	434.76
4.	Warfarin	5 mg tablet (1)	2.41	2.86	3.5	1.31	118.32
5.	Aspirin	75 mg tablet (1)	0.15	0.39	0.65	0.14	178.57
		150 mg tablet (1)	0.23	0.43	0.85	0.14	207.14
		325 mg tablet (1)	0.22	0.25	1.48	0.38	-33.68
6.	Cilostazol	50 mg tablet (1)	5.34	8.39	12.51	5.4	55.46
7.	Clopidogrel	75 mg tablet (1)	2.48	5.41	13.5	1.5	261
8.	Prasugrel	10 mg Tablet (1)	9.48	16.2	143	5.5	194.54
9.	Aspirin + clopidogrel	75 mg + 75 mg tablet (1)	2.37	3.22	7.8	1.8	78.89
10.	Streptokinase	1.5 MIU injection (1)	1,680.08	2,437	3,898.09	812	200.12

Cost variation: essential list versus non-essential list drugs

**Figure 3:**
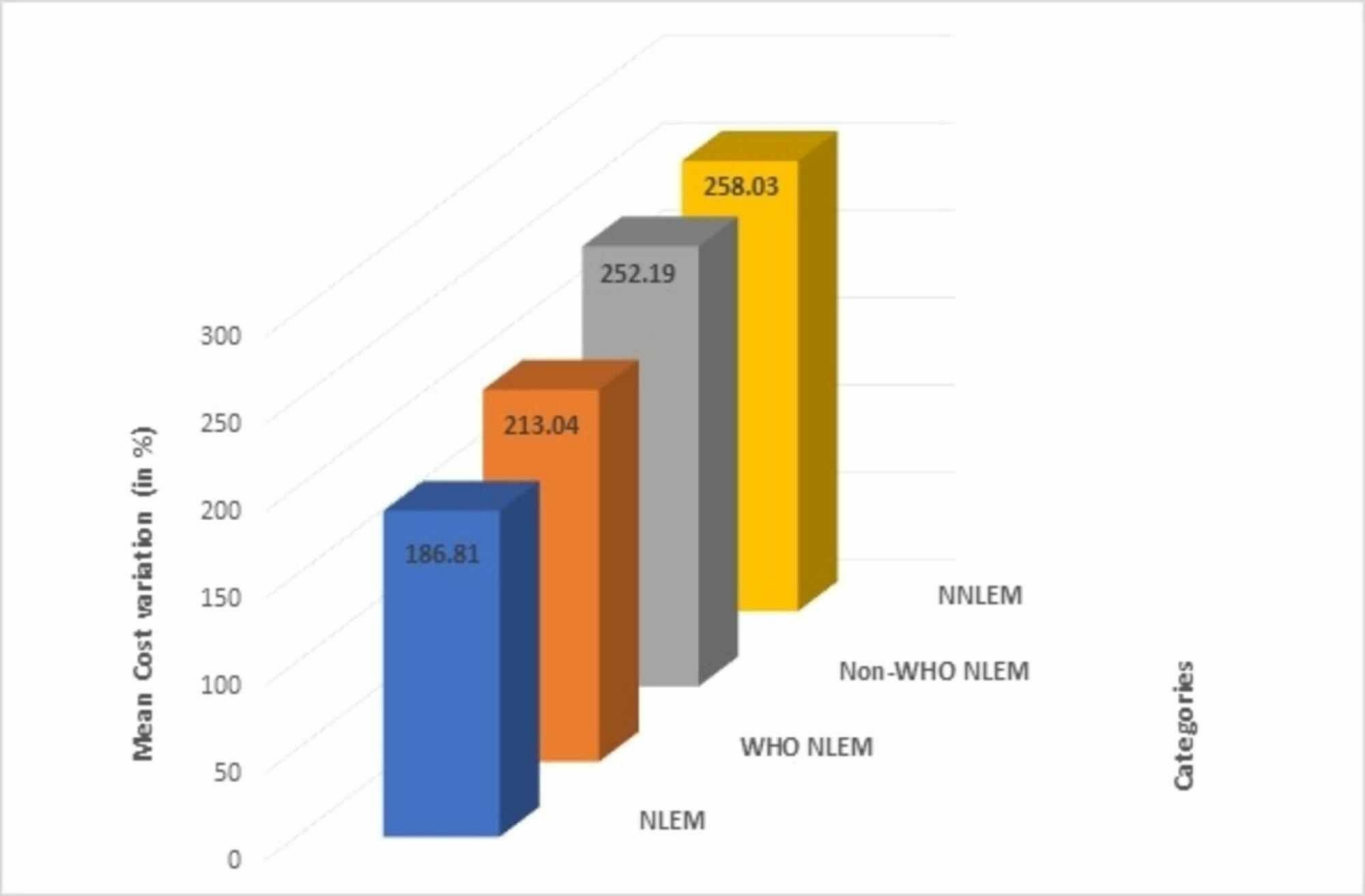
Comparison of mean cost variations among NLEM, NNLEM, WHO NLEM, and Non-WHO NLEM drugs NLEM: National List of Essential Medicines; NNLEM: Non-National List of Essential Medicines; WHO: World Health Organization

The drugs were classified into categories depending on whether they were listed in the NLEM/WHO NLEM or not. The mean cost variation of the NLEM drugs came out to be the least (186.81%) followed by WHO NLEM drugs (213.04%), while it was maximum for NNLEM drugs (258.03%) (Figure 3).

Impact of the selective imposition of price ceiling on cost variation

The selective imposition of price ceiling was observed in a few drugs. For example, while two formulations of enoxaparin, namely 40 mg / 0.4 ml injection and 60 mg / 0.6 ml injection, were under the price control order, 20 mg / 0.2 ml injection was not. The same was observed for different formulations of warfarin, aspirin, clopidogrel, and streptokinase. However, more often than not, the formulations under partial price control had a higher cost variation percentage than their counterparts of the same drug as shown in Tables 1-3.

Impact of the number of available brands on cost variation

**Figure 4:**
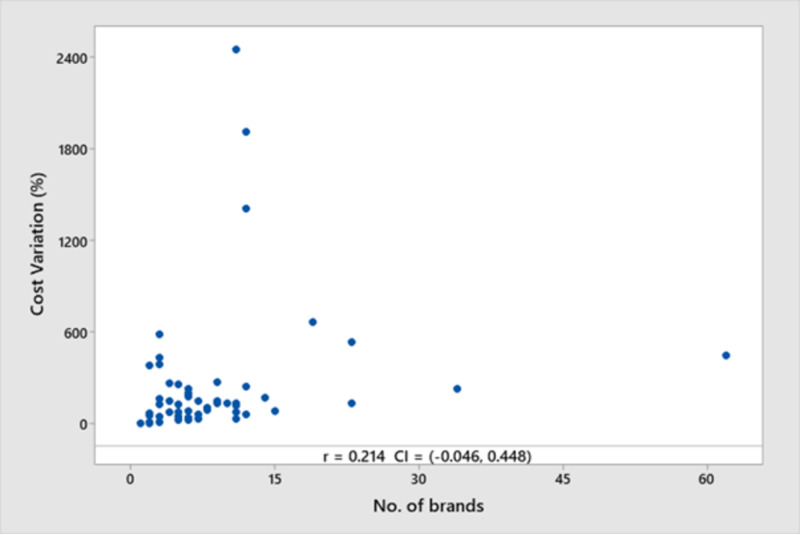
A scatter plot showing the correlation between the number of brands and the cost variation percentage r: coefficient of correlation; CI: confidence interval

Comparison between the number of brands and the cost variation gave a low correlation coefficient (r = 0.214, 95% CI: -0.046 to 0.448, p = 0.106), showing maximum cost variation when the total number of brands was around 12. The ‘r’ value of 0.21 indicated a low degree of association between the two (Figure 4).

## Discussion

Our study showed high cost variation across the spectrum of drugs used for various thromboembolic disorders in India. The findings are in line with other similar studies on various anticoagulants, antiplatelets, and fibrinolytics [14,15]. Besides, such cost variations have been observed previously among other groups of drugs as well [16-18]. All of these serve as evidence to formulate stringent and regularized drug-pricing policies in the nation.

Accessibility to drugs that are considered essential for the management of major diseases remains a yet-to-be-achieved target for most of the low-and-middle-income countries (LMICs). Based on the WHO Model List of Essential Medicines, most of these countries maintain a national list of essential medicines [19]. The challenges associated with the imposition of the price ceiling for essential drugs are higher in these countries as well. This might be due to weak national health systems and policies, poor implementation and monitoring of the national health programs, and other administrative issues. Further, as evident from our results, India does not include important newer drugs such as ticagrelor and prasugrel as well as FDCs such as aspirin + clopidogrel in its essential list and, hence, there is a mismatch between the nation’s health needs and the pharmaceutical policies.

We showed comparative cost variations of some of the most commonly prescribed drugs for thromboembolic disorders in Figure 1. Fondaparinux 2.5 mg / 0.5 ml injection had the lowest cost variation (20%), while it was highest for prasugrel 10 mg tablet (1,408.44%). Figure 2 showed the cost variation of some of the important formulations under DPCO pricing. Streptokinase 1.5 MIU injection showed the highest cost variation (132.02%) while enoxaparin 60 mg / 0.6 ml injection showed the least (4.99%). Table 4 showed the cost variation of some of the important formulations under the JAS scheme. Acenocoumarol 2 mg tablet showed the highest cost variation (680.09%) and cilostazol 50 mg tablet showed the least cost variation (55.46%). Hence, wide variations in drug prices exist across different therapeutic classes, and policies should be implemented to curb them as much as possible. Cost/DDD indicates an estimated expenditure of a patient per day for a particular drug formulation.

The purchase of medicines forms the single largest portion of the total OOP payments by a household in India. It is also estimated that around 20 million people in the nation fall below the poverty line due to health-related expenditures each year [20]. Therefore, the medicines are required to be made available at affordable prices for the masses in general. Drug discovery is an expensive process and most of the pharmaceutical companies try to recover the investments in a short period of time, ignoring the resultant financial burden on the patients.

India had delayed the imposition of drug price control by almost a decade in order to balance the promotion of the domestic pharmaceutical industry and access to essential drugs. The DPCO released in May 2013 after much effort and social activism had a list of 652 formulations for 348 drugs [21]. The NPPA controls the price of the drugs using a market-based mechanism. There were two major policy changes in DPCO 2013, both of which have some issues. Firstly, there is a selective imposition of price control to selective formulations of a particular drug, based mainly on their dose strengths, and excluding other doses and FDCs. This is creating incentives for companies to aggressively market non-regulated formulations of the drugs. Secondly, the ceiling price is based on the simple average of the prices of the top brands of a particular drug in the market having a market share of at least 1%. This also takes into consideration the costlier time-release formulations of the drugs, and if they happen to hold more than 1% of the market share, they will push up the ceiling prices as a whole.

There are several ways to combat these problems. One way is to discard the policy of partial price control and impose price control on all formulations of a drug, especially those which are in NLEM. Another way is to refine the method of determining the ceiling price to make it more effective. The current method allows larger firms to coordinate on prices and evade price regulation. Recently, the Government of India has started collecting price data from individual manufactures. If this process can be sped up, the regulation can become more effective since the firms will not be able to coordinate and push up their prices before the regulation order. India can also update its NLEM more regularly, as done for WHO’s model list, bringing more drugs under price control.

Another alternative is to promote generic medicines. Some studies based on the comparative effectiveness of generic versus brand-name medications have found the clinical outcomes associated with both quite comparable; this should instill more confidence in the patients and healthcare providers on the quality of generic medicines [22,23]. However, in India, patients have a negative perception of generic medicines. Hence, they are often marketed under a brand name. The pharmacists are also not inclined towards selling generic medicines due to low profit margins. Various regulators have suggested that de-branding generic drugs is likely to be more effective than price regulations to bring down the prices of essential drugs. To promote this, legislative measures to mandate doctors to prescribe generic drugs rather than brand names have been taken [24].

The number of medicines under the price control by NPPA is very few and large cost variations are seen among those as well (Figure 2). Nevertheless, we should try and bring more essential medicines under price control and increase the affordability for the masses. Similarly, a huge difference between the generic prices and the median brand prices could be seen among medicines available at the Jan Aushadhi stores under the Pradhan Mantri Jan-Aushadhi Yojana (Table 4). The government should hence promote the practice of prescribing generic medicines and ensure their availability at designated stores at all times. Further, as shown in Figure 3, NLEM drugs have a much lower cost variation than the NNLEM drugs, which indicates that the government should try and include more and more drugs in the NLEM to make medicines more affordable for the masses.

Similar to India, the prices of therapeutically similar drugs in the United States vary widely, which has prompted the policymakers and healthcare insurers to adopt reference pricing. In this, drugs are grouped according to the therapeutic class, and the payment covered by the insurer or the payer is limited to the cheapest or one among the cheapest drugs in that class. Patients who opt for drugs costlier than the reference drug are notified regarding the availability of cheaper alternatives. They are also advised to discuss the alternatives with the prescribing physician. If the physician decides that the reference drug might have a sub-therapeutic response or an adverse reaction, they submit an exception report. The patient has to pay the excess amount (the difference in cost between the preferred and the reference drug). This can be included as a part of medication concordance in regular clinical practice, which is based on shared decision making between the doctor and the patient [25]. Many countries are using this strategy to attenuate increased pharmaceutical spending [26,27].

Most of the treating physicians are often not aware of the prices of branded medicines. They must keep in mind the financial condition of the patient and adopt a shared decision-making approach in prescribing medications. Results from a study have indicated that providing doctors with a manual of comparative drug prices including most of the available brands in the country is associated with a reduction in the patient’s expenditure on medications [28].

The impact of the number of brands of a particular drug formulation on the price was also explored (Figure 4). A weak correlation was found between the two parameters, suggesting that the number of brands does not have a major impact on cost variation. Since this study was focused mainly on commonly used drugs used for treating thromboembolic disorders, this might not always hold true, and further, larger pharmacoeconomic analyses covering other therapeutic classes need to be performed in order to get a broader perspective.

Limitations

Our study has a few limitations. First, we considered a limited number of brands of different drugs as mentioned in CIMS and MIMS even though there are many other brands available in the market. Secondly, the prices of the generic medicines have been obtained from the official website of BPPI, while there are branded generics available in the market whose costs have not been considered. Hence, similar studies should be done on a larger scale based on the same therapeutic class of drugs to overcome these limitations and give us a better picture of cost variation in the Indian pharmaceutical market.

## Conclusions

Our study concludes that a wide variation in pricing is found in the Indian market for various drugs used to treat thromboembolic disorders. Reducing the cost variation and thereby improving the affordability of drugs would improve medication compliance, the health status of the community, and the national economic burden due to healthcare expenditures. Physicians should be made aware of the prices of various drugs while encouraging them to prescribe generic medicines. Emphasis should be laid on expanding the NLEM by including more life-saving medicines, allowing the masses to have access to those drugs as and when needed. Only a collective effort would propel us towards the goal of “Health for All.”
